# A Simple Model of the Rise and Fall of Civilizations

**DOI:** 10.3390/e25091298

**Published:** 2023-09-05

**Authors:** Rickard Nyman, Paul Ormerod, R. Alexander Bentley

**Affiliations:** 1 Centre for Decision Making Uncertainty, University College London, London WC1H 0PY, UK; r.nyman@cs.ucl.ac.uk; 2 Department of Computer Science, University College London, London WC1 0PY, UK; 3 Volterra Partners LLP, London SW9 6DE, UK; 4 College of Emerging and Collaborative Studies, University of Tennessee, Knoxville, TN 37996, USA; rabentley@utk.edu

**Keywords:** complex systems, emergence, neutral selection, innovation, spatial inequality

## Abstract

The literature on the fall of civilizations spans from the archaeology of early state societies to the history of the 20th century. Explanations for the fall of civilizations abound, from general extrinsic causes (drought, warfare) to general intrinsic causes (intergroup competition, socioeconomic inequality, collapse of trade networks) and combinations of these, to case-specific explanations for the specific demise of early state societies. Here, we focus on ancient civilizations, which archaeologists typically define by a set of characteristics including hierarchical organization, standardization of specialized knowledge, occupation and technologies, and hierarchical exchange networks and settlements. We take a general approach, with a model suggesting that state societies arise and dissolve through the same processes of innovation. Drawing on the field of cumulative cultural evolution, we demonstrate a model that replicates the essence of a civilization’s rise and fall, in which agents at various scales—individuals, households, specialist communities, polities—copy each other in an unbiased manner but with varying degrees of institutional memory, invention rate, and propensity to copy locally versus globally. The results, which produce an increasingly extreme hierarchy of success among agents, suggest that civilizations become increasingly vulnerable to even small increases in propensity to copy locally.

## 1. Introduction

Over the past 10,000 years, most of the world’s population has transitioned from egalitarian foraging societies, whose small scale and social norms limited individual power, wealth and prestige [1,2,3] to a global economy in which institutionally facilitated, inherited wealth differs by perhaps ten orders of magnitude from the poorest individuals to the wealthiest elites [4].

A facilitator of this inequality was the rise of state civilizations 5000 years ago and their subsequent cycles of integration and dissolution [5,6,7,8,9,10]. In prehistory, the likely prerequisites for the origins of state societies include intensified agriculture, which not only supported larger populations of increasingly diverse specializations, but also the unequal access to resources that gave rise to inherited wealth inequalities and status hierarchies [4]. Thousands of years before state societies, for example, early Neolithic societies show the beginnings of inter-generational wealth transmission [11,12,13,14] that became an inherent feature of social complexity [15,16].

Theories differ over whether the elite increased inequality, through wealth inheritance, or decreased it, through re-distributive institutions. On one hand, the kin-based authority of chieftains may have facilitated the redistribution of resources, reducing wealth inequality [17,18,19]. On the other hand, or just later in this process, the inheritance of wealth institutionalized differential access to resources, leading to enforced hierarchy, division of labor, and—as early states began to form—authority based on territory rather than kin [8,16,20,21].

What has caused the decline of early state societies? Is there a general principle? Numerous models attribute the fall of early states, as a breakdown in complexity, as typically driven by exogenous events, the deaths of rulers, or demographic change [6,22,23,24]. Another category of models is more general, effectively seeing early states having arisen and collapsed as two sides of the same coin, through the dynamics of competition between elites and commoners [25,26,27].

Here, we take a general approach, with a model suggesting that state societies arise and dissolve through the same processes of innovation. We focus on ancient civilizations, which archaeologists typically define by a set of characteristics including hierarchical organization, standardization of specialized knowledge, occupation and technologies, and hierarchical exchange networks and settlements.

The central process underlying this organization is cumulative cultural evolution [28,29,30,31], or the capacity for human knowledge to build on itself through advancing knowledge of previous generations by a combination of innovation (copying) and invention (creating novelty).

Here, we demonstrate a model that replicates the essence of a civilization’s rise and fall, in which agents at various scales—individuals, households, specialist communities, polities—copy each other in an unbiased manner but with varying degrees of institutional memory, invention rate, and propensity to copy locally versus globally. The results, which produce an increasingly extreme hierarchy of success among agents, suggest that civilizations become increasingly vulnerable to even small increases in propensity to copy locally.

We use a simple agent-based model that replicates both the rise and fall of socioeconomic inequality and hierarchy. The process is parsimonious: agents at various scales—individuals, households, specialist communities, polities—copy each other in an unbiased manner but with varying degree of institutional memory, invention rate, and propensity to copy locally versus globally. This simple process produces an increasingly right-skewed distribution of success among agents, but also makes them increasingly vulnerable to small parameter changes, particularly any increase in the propensity to copy locally rather than globally.

The widely used model of preferential attachment [32,33,34] is a special case of this general model of unbiased copying. New entrants into the model are attached to existing locations with a probability equal to the proportion of previous entrants attached to each location. Under strict preferential attachment, however, once a sufficient number of agents have entered the model, the higher-ranked locations (in terms of the numbers of attached agents) become effectively locked in. As ‘super-stable’ nodes [35] establish themselves, the probability of the second most popular location, say, overtaking the first becomes exceptionally small.

More conducive to change, even at the top, than preferential attachment, our model of cultural evolution incorporates the aspect of invention; with small probability, μ, any agent entering the model can select a location entirely at random. If the agent does not carry this out, then its location is governed by preferential attachment. The variation introduced by invention means that the rankings of locations do not become locked in; the highest-ranked location will, at some point, be overtaken by a location that previously ranked below it [36]. The higher the value of μ, the less time any location will spend at the top rank [37].

To further differentiate from preferential attachment, a memory parameter, *m*, determines how many previous steps of the model are taken into account when determining the probabilities of copying a given location [38]. Preferential attachment, in which all previous time steps are taken into account, would be a case without invention and where the memory is infinite [37].

Empirically, vertical inequality is well defined using measures like income Gini coefficient, even in archaeological contexts [17], whereas heterarchy (also known as horizontal inequality) is more difficult to measure [39]. In economics, the Herfindahl–Hirschman Index (HHI)—equivalent to the Simpson index in ecology—is used to measure the size of firms in relation to the industry which they are in. It is simply the sum of the market shares, expressed as a proportion, of all the firms in the market. It ranges between 1/N, where N is the number of firms in the market, and 1. When HHI is 1, we have the case of pure monopoly, when there is just a single firm in the market. In archaeology, quantitative data are sparse but can include site hierarchies [40] and measures of individual wealth or status from grave contents [17].

## 2. Materials and Methods

We extend a theoretical model that is already highly cited in scientific literature and generate results from it by simulating the model in Julia. The model code is set out in the Supplementary Material file.

The model is that of neutral selection described above. We initially examine the properties of the model when the locations are isolated from each other. Units of fitness accumulate at each location, but no location is able to obtain further units as a result of its connection to any other location.

Table 1 lists the model parameters. In addition to the invention parameter, μ, subsequent introductions to the neutral model have included a memory parameter, *m*, and an affinity parameter, λ, which characterizes how the influence of other locations on the choice made by an agent declines with (physical or social) distance [37,38,41]. The memory parameter, *m*, determines how many previous steps of a model solution are taken into account when new entrants into the model are allocated to the locations using the principle of preferential attachment.

We have a fixed number, *k*, of locations. Units of fitness enter the model and are attached to the locations. The initial number, $N_{0}$, is allocated at random. At each subsequent step, *N* more fitness units enter. These are attached to the locations by the neutral selection model, with invention parameter μ and memory parameter *m*. The number of fitness units attached to each location is modified by a weighted sum of the numbers at other locations, with the weights being defined by λ.

We also report the turnover of the top *y* locations, i.e., the *y* locations that have the most units of fitness. By turnover, we mean the proportion of the total number of steps in any given solution in which at least one of the top *y* locations is replaced by one not in the top *y* in the previous step of the solution.

## 3. Results

We start with the locations placed on a circle and set the distance between each pair equal to 1. We work with 100 locations. At the moment, there is no rule to make a weak location extinct. They all stay in, no matter how few fitness units they attract.

Initially, as noted above, we simulate the special case of the model in which there are no connections between any of the locations. In other words, the parameter λ does not appear.

The model is programmed in Julia and runs multiple solutions in which 100,000 units eventually enter the model. Test runs indicate that 100,000 is more than enough to establish the properties of the model, as the results for any given set of parameters are the same, even if 1 million or 10 million enter.

We report results for ten integer values of the parameter m from 1 through 10 and for values of twenty values of μ ranging from 0.001 to 0.1. We therefore consider 200 separate pairs of parameter values. We report results after 1000 simulation steps per solution of each parameter pair, which summarizes 500 separate solutions of the model at each time step. Investigation of the model showed that with this number of steps, the results are the same across a range of model properties as when 100,000 or even 1 million steps are used.

The choice of $N_{0}$ and *N* does not substantially affect the results that are obtained. We therefore propose to use $N_{0}=N=100$, although we note that increasing or decreasing *N* during the simulation would favour the most recent and oldest agents, respectively [42].

**Figure 1:**
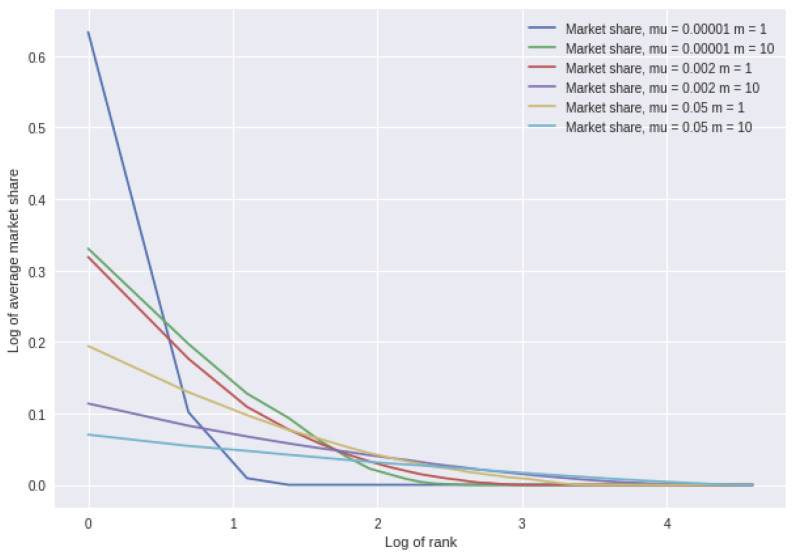
Average market share over 500 simulations as a function of the rank on a log–log scale (note on a log scale, 0 corresponds to rank 1).

The properties of the basic model of neutral choice are well known. By way of illustration, Figure 1 shows some results for various combinations of the parameters µ and m. We plot the average market share of the locations by rank across 500 simulations, where rank 1 is the highest market share and 100 the lowest in each simulation. We use a log–log scale, which illustrates the differences more clearly.

We calculate a range of metrics to describe the output of the model. We measure the degree of concentration of the fitness units by the HH index (HHI), described above. An HHI value of 1 means that one unit has all the fitness. Next, we calculate across all solutions and all steps the time spent at the number 1 position by the location with the most fitness units. We record the top *y* (usually *y* is set to 3 out of the total of 100) and measure the turnover at each step. If the top *y* remains the same as in the previous step, even if they change ranks between each other, no turnover is recorded. We measure the percentage of steps in which turnover is 0, 1, 2, and 3.

When a location stops being number 1, we record the distance from it of the location that replaces it, and the frequency of this distribution (we have to adjust the numbers for location at distance 10, because for any location, there are two at distance 1 through 9, but only one at 10). We also record how many times a location returns to number 1 once it has stopped being number 1. Finally, we measure the length of time it takes for a number 1, once it stops being 1, to drop to rank *y*, where integer *y* ranges from 2 to 20.

We initially examine the properties of the model when the locations are isolated from each other. Units of fitness accumulate at each location, but no location is able to obtain further units as a result of its connection to any other location.

**Figure 2:**
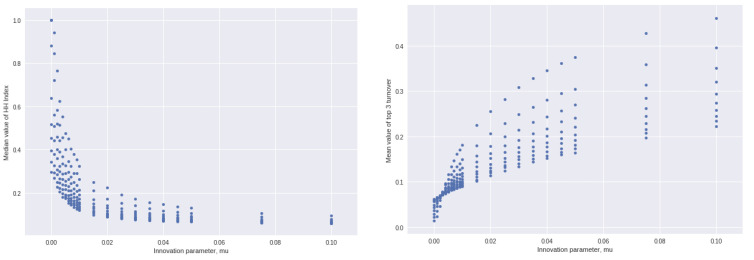
**Left**: Median value of HH index over 500 separate solutions of each step after 1000 steps and the parameter μ, no influence between locations. **Right**: Effect of the parameter μ on the proportion of steps in which just one of the top 3 location drops is replaced by one outside the top 3 in the previous step. Average proportion of steps, across 500 solutions, each of 1000 steps.

Figure 2, left, plots the median value of the HH Index against the parameter *µ*. There is obviously a highly non-linear relationship. The lower the ability of locations to innovate of their own accord, the more concentrated fitness becomes, approaching the theoretical limit of all fitness units being concentrated at a single location as μ approaches zero.

Figure 2, right, shows the effect of μ on the proportion of steps in which just one of the top three locations (in terms of fitness units they have) is replaced by one outside the top three in the previous step. Again, we see a non-linear relationship. As the capacity of locations to innovate of their own accord increases, turnover in the top three rises.

**Figure 3:**
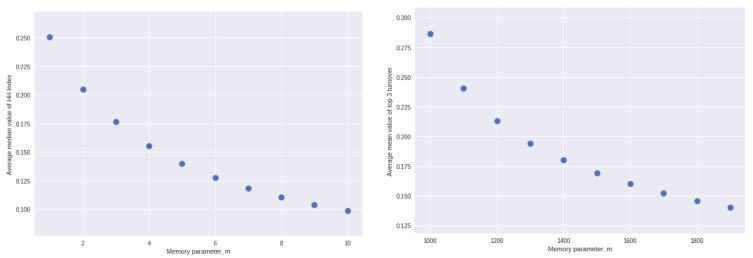
**Left**: Average value of the HH index for each value of the parameter *m* across 500 separate solutions). **Right**: Average proportion of times 1 of the top 3 drops out for each value of the parameter *m* across 500 separate solutions).

There is more variation across the individual solutions when we consider the relationship between the two outcomes of interest and the parameter *m*. Accordingly, we plot in Figure 3 the average values of HHI and turnover, respectively, for each integer value of the memory parameter, *m*.

Table 2 shows estimates, by simple regression, of the logs of both the median HH index and the turnover variable against the logs of the two parameters, μ and *m* (we use logarithms due to the non-linear relationship).

Before carrying out a full analysis of the properties of the model when the affinity parameter, λ, is introduced, we examine the impact of λ when μ=0 and m=1. As noted above, without λ, the solution converges on a ‘winner takes all’ scenario, in which the the HH index is 1 and the turnover in the top three becomes zero.

The values of λ in themselves have no intrinsic meaning. We define the impact of λ through the following formula:

(1) $$w_{ij}=\exp\left(-\lambda d_{ij}^{2}\right)$$

where the distance $d_{ij}$ is the distance between locations *i* and *j* along the arc of a unit circle. Locations are placed equidistantly on the circle.

What is of interest is the weights assigned to each location in terms of its distance from any other. For the values of λ that we use, effectively only the nearest neighbours (i.e., those immediately either side of any particular location) of any given location carry any weight. We examine 28 separate values of the parameter, with the weights on the immediate neighbours varying between 0.0002 and 0.082. We find that for values of λ that assign greater weights, the influence of the parameter becomes very strong, and the distribution of the units of fitness at each location converges on the random uniform.

With no similarity influence or invention, one location obtains all the units of fitness, and the maximum length of time at which any particular location occupies this number 1 slot approaches the number of solution steps of the model (i.e., the winner gets locked in quickly). The effect of affinity λ on the HHI (median after 1000 steps of the model, solved 500 times each step) appears to reach a tipping point, beyond which λ exercises an increasing influence (low lambda means high “influence”). For high vales of λ (lower influence), median HH is always 1.

In terms of turnover in the top three, λ plays a big role, but note that it starts to take effect only when there is more influence than when HH begins to change. This indicates a range in which there is stability in rankings but with a more egalitarian outcome overall. Not surprisingly, the weaker the influence of the number 1 unit, the more likely number 1 is to be replaced by its immediate neighbours. These results are illustrated in Table 3 and Table 4, which show the regressions of HHI and turnover on μ and *m* for selected values of λ.

**Figure 4:**
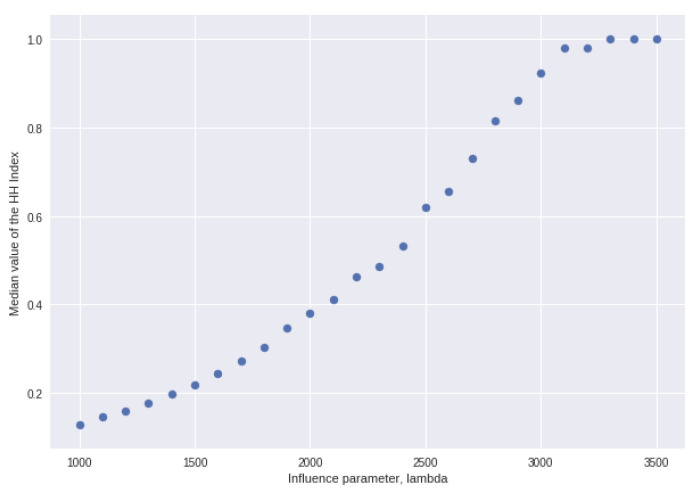
Median HH index after 1000 steps and 500 separate solutions and the influence parameter λ, with μ=0 and m=1.

In all the regressions of Table 3 and Table 4, the estimated coefficients are highly significantly different from zero. Figure 4 plots the median value of the HH index, again after 1000 steps of the model and 500 separate solutions, and λ, with μ=0 and m=1 in all solutions. For values of λ>4000, in other words, when influence is weak, after 1000 steps in all solutions, the value of HHI has converged on its theoretical value of 1. Figure 4 therefore shows the effect of values of λ in the range 1000 to 3500.

A simple linear regression of the HHI on lambda gives ${\bar{R}}^{2}=0.967$. Similarly, a linear regression of the proportion of steps in which one of the top three locations is replaced on λ yields ${\bar{R}}^{2}=0.855$.

When λ is introduced to the model, there is a third statistic in the output that is of interest. When top location is replaced as the top by another, we measure the proportion of these events in which it is replaced by either of its immediate neighbours, by those next to them and so on to the location furthest away. As the influence of λ declines, the replacement becomes more and more random with respect to distance (Table 5).

Finally, we simulate the model with the previous 200 combinations of *m* and μ (μ>0) with 28 finely graded values of λ, making 5600 combinations in total.

A linear regression of the median value of HHI after 1000 steps with 500 separate solutions at each step on the three parameters (all variables in natural logs) gives and ${\bar{R}}^{2}=0.882$. Carrying out the linear regression for each of the 28 values of λ increases this to ${\bar{R}}^{2}=0.975$. Similarly for turnover, we obtain ${\bar{R}}^{2}=0.883$ and ${\bar{R}}^{2}=0.958$, respectively.

Using machine learning algorithms, we can obtain an almost perfect fit to the values in the model solutions. We carry out 5-fold cross-validation and report the fits for the out of sample “predictions”. Using the random forest algorithm in Python and the default values of the required inputs yields ${\bar{R}}^{2}=0.996$ for HHI and ${\bar{R}}^{2}=0.999$ for turnover.

The machine learning output gives an estimate of feature importance. This is not, to put it into the more familiar context of linear regression, whether a particular variable has a coefficient that is significantly greater than zero, but how much it contributes to the overall fit. For the HHI, for example, the most important is invention rate, μ, with *m* and λ being very similar. This finding is reflected in linear regression, in which each of the three factors is left out in turn.

From the above analysis, it will be apparent that the model, with its three parameters, can readily be calibrated to a wide range of empirical non-Gaussian outcomes. By way of illustration, we can usefully consider the degree of inequality that existed in early societies, for which a large number of estimates have been made.

In archaeology and anthropology, the distributions of site sizes, house sizes, burial wealth, or individual wealth, for example, are often estimated from sparse data. For this reason, a useful metric is the Gini coefficient [17]. Table 6 shows how the median value of the Gini relates to the three model parameters by simple linear regression. All parameters have a significant effect. As expected [37], invention reduces the Gini coefficient because the introduction of variation increases turnover among the top ranks. Increasing the memory parameter readily reduces the Gini coefficient, because memory acts to retain variation, as less copied locations are not as quickly ‘forgotten’.

The effect of the affinity parameter λ is to increase Gini coefficient. It is important to note that a higher λ means a steeper decline in interaction with distance. So, the less the interaction between locations is, the greater the degree of inequality between them (other things being equal). Of course, the lower the level of interaction, the lower the level of competition that the leading sites face, so the impact of the parameter is in line with theoretical expectations.

With the parameter triples that we described above, the median value of the Gini coefficient across all the solutions for each of the individual triples ranges from 0.36 to as high as 0.99. It is straightforward, however, to obtain lower values by widening the range of parameters. For example, increasing memory reduces the Gini coefficient.

## 4. Discussion and Conclusions

Overall, the results demonstrate the capacity of the model to be calibrated against a wide range of different outcomes. It offers a theoretical framework for the understanding of the rise and fall of civilisations. The parsimonious model containing only the invention rate and memory tends to produce not only low turnover amongst the top-ranked locations in terms of fitness units but, especially for low values of invention, to exhibit “winner-take-all” properties. These properties, as noted in the Introduction, are typical features of early states, in which secrecy and hierarchy were important.

The parameter λ has a significant effect on the model, and relates to the many approaches in archaeology to how network interactions facilitated the rise of early states and the specialization, hierarchy/heterarchy, and inequality.

Our model can readily generate a very broad range of Gini coefficients that represent a range of sociopolitical economies from broadly egalitarian to highly unequal. The degree of inequality within a given society at a point in time can readily be calibrated using the model. The different stages of its evolution will be characterised by different values of the three parameters of innovation, memory, and affinity.

Substantial literature exists that provides estimates on the degree of inequality within early societies [17,43]. Among modern hunter-gatherers, for instance, the Gini coefficient is around 0.17 [4]. Gini coefficients are estimated between 0.35 and 0.46 in early farming societies [4] as resource access began to be inherited over generations [12]. In early complex state societies, estimated Gini coefficients include 0.57 at Cahokia (Mississippian N. America), 0.53 at Dadiwan in Late Yangshao China, 0.52–0.54 at Herculaneum and Pompeii in the Roman Empire, 0.62 at Mayan Tikal, and 0.68 at Kahun in Middle Kingdom Egypt [4].

**Figure 5:**
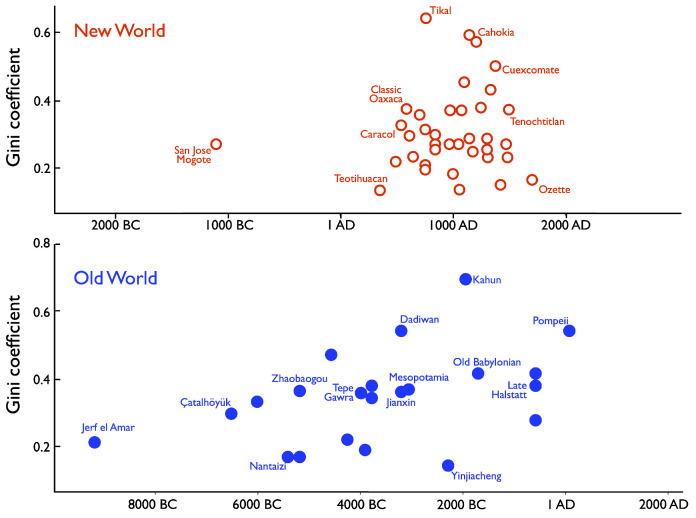
Gini indices through time in ancient societies. After [4].

From the perspective of offering initial evidence for the ability of the model to be calibrated to data, a key point to note is that inequality within any given society was in general not time-invariant. In prehistory, inequality generally increased over time. In prehistoric western North America, for example, analysis of the grave goods in hundreds of burials spanning several millennia indicate that Gini index increased from about 0.3 to about 0.6 [17,44]. More broadly, Figure 5 shows how Gini indices in ancient societies of the Old World and New World changed over thousands of years, using house-size distributions [4], which can be a good proxy for wealth [17]. In the New World, a decline in Gini index occurred upon the collapse of Mayan states around AD 900, even as it increased in North America into the Mississippian, at Cahokia (Figure 5).

The difference between Teotihuacan, where the estimated Gini index is quite low at 0.12, versus the Mayan polity of Tikal with its high Gini index of 0.62 [4], suggests the importance of the affinity parameter, λ, as a measure of social distance. An established archaeological model of how elites maintain power [45,46] describes network strategies, as in Maya societies, which emphasize long-distance exchange with other elites to maintain their elite social ties and obtain exotic goods used to reward their followers. Although items are exchanged over geographic distances, the elite social network was exclusive, and hence, λ would be high. In contrast, leaders in corporate strategies, as at Teotihuacan, derived power from locals living and working in the city or region [17,45,47]. Although these people were close by, their ranks were more diverse, and hence, λ would be smaller.

In other words, the elite social network strategy is analogous to high λ, whereas the local kin-based corporate strategy resembles a low λ. The way that λ predicts HHI in the simulations seems to reflect this ‘corporate-network’ distinction. In the corporate strategy, elites facilitate the redistribution of wealth [18,19]. Through kin-based authority, early elites were facilitators of economic cooperation and levellers of inequality—‘bankers’ who managed the local economy to everyone’s benefit [48]. Early states varied in their degree of redistribution, from temple-based redistributive states (e.g., Mesopotamia) to subsequent expansive and tributary states where wealth and land, as war spoils, were accumulated by state elites, not for redistribution [20,21,49]. This accumulation required a hierarchy of people in a bureaucracy as well as settlements, from producers to assembly points to central points for redistribution [22]. As societies became more complex, the network strategy likely predominated, as elites competed to hold power, increasingly through exclusive, long-distance exchange between elites of other societies rather than merely support of local populations. Examples include the Classic Maya society, or even the 9th-10th century trade of prestigious gifts at the highest levels of social and political power across the Islamic Mediterranean world, from Western Europe to China [50]. State institutions and systematized differential access to resources, enforced hierarchy, division of labour, and authority were based on territory rather than kin [8,17,21].

In a more general way, archaeologists often emphasize the interdependence of specialization and intergroup cooperation, often called “heterarchy” [8,17,21,47,51] or ‘horizontal inequality’ [9,52]. In small-scale societies, intergroup competition often yields asymmetric payoffs that differentiate groups in terms of status, wealth, ethnic group [53], which can motivate those profiting from such inequality to initiate between-group aggression, even at the expense of the welfare of their group [54].

An example is the Indus civilization, a homogeneous 500,000 square miles of standardized crafts, which measures cities and urban planning, with a four-tier settlement hierarchy. Lacking rich tombs or elite residences, there is little evidence that the Indus civilization was highly socially stratified; instead, the Indus Valley civilization reflects heterarchy through a sorting of the population by craft and settlement specialization, facilitated by the administrative and economic functions of writings and seals. Cities like Mohenjo–Daro and Harappa grew because they lay at the crossroads within trade networks that connected different agriculture and resource zones [7]. Certain merchants may have profited as ‘hubs’ in this network, by controlling the knowledge and multi-step specialized production of complex technology like faience, stoneware, and red carnelian and other long-distance exchange goods [7,55]. Around 1900 BCE, the core cities of the Indus collapsed and were abandoned, with the disappearance of urban forms, public buildings, and the written script. This has been interpreted through both exogenous and endogenous causes, such as a well-documented regional drought [56] that disrupted the trade networks, which then destabilized the traditional structures of authority within the Indus civilization.

In principle, our model is applicable to contemporary societies. Models of modern economic inequality invoke bad governance, political instability, and conflict from outside groups [5,9], whereas other models emphasise intrinsic causes such as the increase in political and economic inequality to the point of driving grievances between competing groups within the larger society [57,58,59,60,61].

## Figures and Tables

**Table 1:** Parameters of the model.

Parameter	Description	Analogical Examples
μ	Invention rate	Novel inventions, organizations, or settlements
*m*	Memory	Oral tradition, cultural memory, written records
λ	Affinity	Trade networks, geographic distance, social distance
*N*	Agents	Population size or number of organizations

**Table 2:** Regressions of HHI and turnover (of 1 of the top 3) against the parameters μ and *m* (all variables in natural logs).

Statistic	log (Median HHI)	log (Turnover)
Intercept	−3.281	0.022
Standard error	0.028	0.045
Coeff. onlog(μ) (s.e.)	−0.471 (0.005)	0.393 (0.008)
Standard error	0.005	0.008
Coeff. onlog(m) (s.e.)	−0.406 (0.009)	−0.230 (0.015)
${\bar{R}}^{2}$	0.979	0.922
Residual std. error	0.093	0.148

**Table 3:** Regressions of HHI and turnover of 1 of the top 3 on μ and *m*, selected values of λ, all variables in natural logs. The dependent variable is HHI (median value, in logs).

λ	μ	*m*	${\bar{R}}^{2}$
1000	−0.077	−0.225	0.960
2000	−0.253	−0.354	0.974
3000	−0.424	−0.395	0.979
3500	−0.455	−0.405	0.980
4000	−0.468	−0.405	0.980
5000	−0.471	−0.407	0.979

**Table 4:** Regressions of turnover of 1 of the top 3 on μ and *m*, selected values of λ, all variables in natural logs. The dependent variable is the proportion of times that one of the top three turns over (in logs).

λ	μ	*m*	${\bar{R}}^{2}$
1000	0.084	0.312	0.966
2000	0.233	−0.324	0.968
3000	0.353	−0.266	0.949
3500	0.378	−0.245	0.934
4000	0.389	−0.236	0.927
5000	0.392	−0.231	0.923

**Table 5:** Regression of the proportion of times that number 1 is replaced with an immediate neighbour, versus selected values of μ, *m*, and λ. All variables are listed as natural logs (n.s. means not significantly different from zero).

λ	μ	*m*	${\bar{R}}^{2}$
1000	−0.148	−0.048	0.888
2000	−0.225	−0.132	0.918
3000	−0.088	−0.168	0.643
3500	−0.029	−0.050	0.402
4000	−0.008	−0.017	0.105
5000	n.s.	n.s.	∼0

**Table 6:** Regressions between median Gini index and three model parameters. Adjusted $R^{2}$ = 0.871.

	Estimate	Std. Error	t Value	Pr(>|t|)
Intercept	0.89	0.0022	397.3	<0.0001
μ	−3.76	0.024	−158.5	<0.0001
*m*	−0.0202	0.0002	−94.6	<0.0001
λ	0.00000215	0.00000003	74.0	<0.0001

## Data Availability

The Julia code for this study is provided in the Supplementary Materials.

## Supplementary Materials

The Julia code for this study can be downloaded at: https://www.mdpi.com/article/10.3390/e25091298/s1.
