# Supplementary material for: A Simple Model of the Rise and Fall of Civilizations
Source: Entropy (Basel). 2023 Sep 5;25(9):1298. doi: 10.3390/e25091298 (PMC10529410; doi:10.3390/e25091298)
Supplement: Supplementary file 1 [file entropy-25-01298-s001.zip › entropy-2524101-Supplementary.pdf]

Supplementary Materials: "A Simple Model of the Rise and Fall of Civilizations"

Rickard Nyman, Paul Ormerod and R. Alexander Bentley

Julia code for model:

```
@everywhere using Distributions
#using Plots
@everywhere using StatsBase
@everywhere using DataStructures
@everywhere using Profile
@everywhere using DataFrames
@everywhere using Statistics
#using PProf

@everywhere function getPopGrowth(r::Float64,
                        num_steps::Int, P::Int, C::Int)
    vals = zeros(num_steps)
    for step = 1:num_steps
        change = ceil(r * P * (1 - P / C))
        vals[step] = change
        P += change
    end
    return vals
end

@everywhere function shiftProdArray(a::Array{Int}, m::Int)
    for k = m:-1:2 #reverse(2:m)
        a[k] = a[k-1]
    end
    a[1] = 0
    return a
end

@everywhere function sampleProductWeighted(P::Int,
            weights::Array{Float64}, num::Int)
    return sample(1:P, Weights(weights), num)
end

@everywhere function sampleProduct(P::Int, num::Int)
    return sample(1:P, num)
end

#@everywhere const popGrowth = getPopGrowth(0.001, 7400,
                                100, 1000000)


function getDistanceMatrix(λ)
    distances = zeros(20,20)
    for i=1:20
        for j=1:20
            arc = abs(i-j)/20
```

```
            distances[i,j]
                =round(exp(- λ * (minimum([arc, 1-arc])^2)),
                        digits=4)
        end
    end
    return distances
end

function getDistanceMatrix(λ, num_locations)
    distances = zeros(num_locations,num_locations)
    for i=1:num_locations
        for j=1:num_locations
            arc = abs(i-j)/num_locations
            distances[i,j]=exp(−λ * (minimum([arc, 1-arc])^2))
        end
    end
    return distances
end

@everywhere function model_run(μ, m, λ, num_steps,
        agents_each_step, num_locations)

    distanceMat = getDistanceMatrix(λ, num_locations)
    #distanceMat = interpolate(λ, num_locations)
    locations = zeros(num_steps + m, num_locations)
    locations_sum = zeros(num_locations)
    location_weights = [1 / num_locations for i = 1:num_locations]
    ranks ::Matrix{Float64} = zeros(num_steps, num_locations)
    ranksCorrelation::Array{Float64} = zeros(num_steps)
    market_share::Matrix{Float64} = zeros(num_steps, num_locations)
    counts::Matrix{Float64} = zeros(num_steps, num_locations)
    shocked_index::Array{Float64} = zeros(num_steps)
    #market_share_final:: Array{Float64} = zeros(num_steps, 20)

    for s = 1:num_steps

        # number of agents to innovate (sample with equal weights)
        innovate_agents = 0
        for i = 1:agents_each_step
            if rand() <= μ
                innovate_agents += 1
            end
        end

        for id = sampleProduct(num_locations, innovate_agents)
            locations[(s+m), id] += 1
        end

        for id = sampleProductWeighted(num_locations,
            location_weights, agents_each_step
                    - innovate_agents)
#sample(1:model.P, Weights(model.weights),
                    Int(to_add)-innovate_agents)
            locations[(s+m), id] += 1
```

```
    end

    for i = 1:num_locations
        locations_sum[i] = sum(locations[(s):(s+m), i])
    end


    for i = 1:num_locations
        weight_sum_i = locations_sum[i]
        for j = 1:num_locations
            if i!=j
                weight_sum_i+=locations_sum[j]
                        * distanceMat[i,j]
            end
        end
        location_weights[i]=weight_sum_i
    end

    # adjust the location_weights by shock proportion
    #for all connected locations
    # probability of shock to one of the 20 locations
    shocked_i = sample(1:num_locations)
    shocked_index[s] = shocked_i
    shock_proportion = rand(Uniform(0.5,1))

    for i = 1:num_locations
        # adjust i by subtracting propagated shock
        # proportion by distance to shocked_i
        location_weights[i]=location_weights[i]
        -location_weights[i]
            *shock_proportion*distanceMat[i, shocked_i]
    end

    # locations weights is weighted sum of
    #location_sums (by distance matrix)
    counts[s,:] = location_weights
    total = sum(location_weights)
    location_weights /= total
    market_share[s,:] = locations_sum / sum(locations_sum)
    ranks[s,:] = competerank(market_share[s,:], rev=true)
    if s > 1
        ranksCorrelation[s]
            = corspearman(ranks[s,:], ranks[s-1,:])
    end
    #market_share_final[s,:]
            = sort(market_share[s,:], rev=true)
end

return DataFrame(ranks=[ranks[i,:] for i in 1:size(ranks,1)],
corr=ranksCorrelation,
market_share=[market_share[i,:] for i
      in 1:size(market_share,1)],
counts=[counts[i,:]
      for i in 1:size(counts,1)], shocked_index=shocked_index)
```

```
end

# this is the one we use
function runSim(μ::Float64, m::Int, λ, num_steps,
             agents_each_step, num_sims,
    num_locations)
    HH = Array{Float64}(undef, num_sims)
    #convert(SharedArray{Float64,1},
    Array{Float64}(undef, 100))
    #MaxRank1 = Array{Float64}(undef, 100)
    turnover_k = Array{Float64}(undef, num_sims, 4)
    rank1counter = Array{Vector{Int}}(undef, num_sims)
    dist_rank1_change = Array{Vector{Int}}(undef, num_sims)
    backto1 = Array{Float64}(undef, num_sims)
    how_long_back1 = Array{Vector{Int}}(undef, num_sims)
    shock_overturn_1_count = 0
    market_share_final = Array{Float64}(undef,
                    num_sims, num_locations)
    #market_share = Array{Vector{Float64}}(undef, 100)
    for i = 1:num_sims
        out = model_run(μ, m, λ, num_steps,
                agents_each_step, num_locations)
        HH[i] = sum(out.market_share[num_steps,:][1].^2)
        ranks = reduce(hcat,out.ranks)'
        counter_times_return1 = 0
        backRank1 = zeros(0)
        shocked_index = out.shocked_index

        for j=1:num_locations
            istop = ranks[:,j] .== 1
            back1 = (istop[2:num_steps]
                     - istop[1:(num_steps-1)]) .== 1
            how_often_back = sum(back1)
            if how_often_back > 1
                when_back = findall(==(1), back1)
                append!(backRank1, when_back[2:how_often_back]
                -when_back[1:how_often_back-1])
            end
            counter_times_return1 += max(0, how_often_back-1)
        end

        how_long_back1[i] = backRank1
        backto1[i] = counter_times_return1 #./ (num_steps*20)
        #market_share[i] = out.
        #MaxRank1[i] = maximum(count.(==(1.0),
        #         eachcol(ranks))./num_steps)
        atRank1 = zeros(0)
        rank1_change = zeros(0)
        count_rank1 = 1
        turnover = [0,0,0,0]
        # vector to count turnover in top k (=3) from t-1 to t
        topk = Set([1, 2, 3])
        for j=2:num_steps
            topknow = findall(x -> x in topk, ranks[j,:])
```

```
                topkprev = findall(x -> x in topk, ranks[(j-1),:])
                common_elements = minimum([3,
                        length(intersect(topknow,topkprev))])

                turnover_num = 3-common_elements
                turnover[(turnover_num+1)] += 1

                topnow = findall(x -> x == 1.0, ranks[j,:])
                topprev =  findall(x -> x == 1.0, ranks[(j-1),:])

                #check if topprev got shocked and is no longer top
                #println("shocked index:", shocked_index[j-1])
                #println("top prev:", topprev)
                #println("")
                for t in topprev
                    if t == shocked_index[j-1]
                        #for k in topprev
                        if !(t in topnow)
                            shock_overturn_1_count += 1
                        end
                    end
                end

                # if the same agent in the top spot,
                # increment the counter
                if topnow == topprev
                    count_rank1 += 1
                else
                    # new agent in top spot
                    append!(atRank1, count_rank1)
                    if topnow[1] != topprev[1]
                        append!(rank1_change,
                        minimum([abs(topnow[1]-topprev[1]),
                        num_locations-abs(topnow[1]-topprev[1])]))
                    end
                    #append!(rank1_change, abs(topnow[1]-topprev[1]))
                    count_rank1=1
                end
            end
            append!(atRank1, count_rank1)
            turnover_k[i,:] = turnover./(num_steps-1)
            rank1counter[i] = atRank1
            dist_rank1_change[i] = rank1_change
            #println(sort(out.market_share[num_steps]))
            market_share_final[i,:] =
                sort(out.market_share[num_steps], rev=true)
            #println(sort(market_share_final[i,:]))
        end
        return [HH, rank1counter, turnover_k,dist_rank1_change,
                backto1,how_long_back1, shock_overturn_1_count,
                market_share_final]
end

using Serialization
```

```
using FreqTables

num_locations = 100
header = vcat(["mu", "m", "lambda", "hh_min", "hh_1q",
"hh_med", "hh_mean",  "hh_3q", "hh_max", "num1_min", "num1_1q",
    "num1_med", "num1_mean", "num1_3q", "num1_max",
    "turn0_mean", "turn1_mean", "turn2_mean", "turn3_mean"],
    ["repdist_" * string(i) for i =1:Int(num_locations/2)],
    ["time_back1_min", "time_back1_1q", "time_back1_med",
    "time_back1_mean", "time_back1_3q", "time_back1_max",
    "back1_min", "back1_1q", "back1_med", "back1_mean",
    "back1_3q", "back1_max", "shock_overturn_1_count"],
    ["market_share_final_" * string(i)
                    for i=1:(num_locations * 100)])


function describeStats(row :: Array{Float64})
    stat = nquantile(row, 4)
    return [stat[1], stat[2], stat[3], mean(row), stat[4], stat[5]]
end

function describeStats(row :: Array{Int})
    stat = nquantile(row, 4)
    return [stat[1], stat[2], stat[3], mean(row), stat[4], stat[5]]
end


## select ranges of parameters to run the model over

#m_s = [1] #[1,2,3,4,5,6,7,8,9,10]
#m_s = [1,2,3,4,5,6,7,8,9,10]
m_s=[1, 10]
#m_s = [1,2,3]
#μ_s = [0.0] # [0.0, 0.001,0.002,0.003,0.004,0.01,0.015,
         0.02,0.025,0.03,0.035,0.04,0.045,0.05,0.075,0.1]
#μ_s = [0.0, 0.001,0.002,0.003,0.004,0.005, 0.006, 0.007,
         0.008,0.009,0.01,0.015,0.02,0.025,
         0.03,0.035,0.04,0.045,0.05,0.075,0.1]
#μ_s = [0.00001, 0.02, 0.05]
μ_s = [0.00001, 0.02, 0.05]


lambda_s = vcat(collect(1000:100:3500), [ 4000, 5000])


function getLambda(x)
    return -log(x) / (1/100)^2
end


equivalent_lambda = []
for lambda = lambda_s
    dist_mat = getDistanceMatrix(lambda)
    push!(equivalent_lambda, getLambda(dist_mat[1,2]))
```

```
end

equivalent_lambda[27]=100000
equivalent_lambda[28]=100000


lambda_s = equivalent_lambda
#lambda_s = vcat(collect(1000:100:3500), [ 4000, 5000])
#lambda_s = vcat(collect(2500:100:3500), [ 4000, 5000])


DATA = Vector{Float64}[]

count_param_run = 0
num_sim = 100
for μ in μ_s
    for m in m_s
        for lambda in lambda_s
            res = runSim(μ, m-1, lambda,
            1000, 100, num_sim, num_locations)

            seqs = Vector{Float64}()
            for i=1:num_sim
                seqs=vcat(seqs, res[2][i])
            end

            seqs2 = Vector{Float64}()
            for i=1:num_sim
                seqs2=vcat(seqs2, res[4][i])
            end

            seqs3 = Vector{Float64}()
            for i=1:num_sim
                seqs3=vcat(seqs3, res[6][i])
            end

            row = zeros(0)
            append!(row, describeStats(res[1]))
            append!(row, describeStats(seqs))
            append!(row, [mean(res[3][:,1]), mean(res[3][:,2]),
            mean(res[3][:,3]), mean(res[3][:,4])])
            tbl = freqtable(seqs2)
            prp = prop(tbl)
            prp[1:Int(num_locations/2 - 1)] /= 2
            append!(row, prp)
            append!(row, describeStats(seqs3))
            append!(row, describeStats(res[5]))
            append!(row, res[7])
            #println(res[8])

            #append!(row, [mean(res[8][:,i])
                    for i=1:num_locations])

            for i=1:num_locations
```

```
                append!(row, res[8][:,i])
            end

            prepend!(row, [μ, m, lambda])

            push!(DATA, row)
            count_param_run += 1
            if mod(count_param_run, 100) == 0
                print("Run ")
                print(count_param_run)
                print(" parameter combinations \n")
            end
        end
    end
end

using DataFrames

DATA_t = collect(eachrow(reduce(hcat, DATA)))
df = DataFrame(DATA_t, header)

using CSV
#CSV.write("test.csv", df)

CSV.write("ModelStats.csv", df)
```
